# A Rare Consequence after Shoulder Dislocation in a Professional Cyclist: A Case Report

**DOI:** 10.3390/medicina55090529

**Published:** 2019-08-25

**Authors:** Claudio Ceccarelli, Fabrizio Brindisino, Mattia Salomon, John Duane Heick, Filippo Maselli

**Affiliations:** 1 Physiotherapy and Manual Therapy, 54100 Massa, Italy; 2 Department of Medicine and Health Science “Vincenzo Tiberio”, University of Molise, 86100 Campobasso, Italy; 3 FTM, Physiotherapy and Manual Therapy, Physiotherapy Department, 73100 Lecce, Italy; 4 CST Trento S.r.l. Physiotherapy Department, 38121 Trento, Italy; 5 Department of Physical Therapy and Athletic Training, Northern Arizona University Flagstaff, Flagstaff, AZ 86011, USA; 6 Department of Neuroscience, Rehabilitation, Ophthalmology, Genetics, Maternal and Child Health, University of Genoa-Campus of Savona, 17100 Savona, Italy; 7 Sovrintendenza Sanitaria Regionale Puglia INAIL, 70124 Bari, Italy

**Keywords:** cycling, shoulder dislocation, dystonia, physical therapist’s differential diagnosis, referral

## Abstract

*Background:* Cycling is a popular source of recreation and physical activity for children and adults. With regard to the total number of sports injuries, cycling has the highest absolute number of injuries per year in the United States population. Cycling injuries can be classified into bicycle contact, traumatic, or overuse injuries. *Aim of this study:* The aims of this case report are to report a rare clinical complication of glenohumeral joint anterior dislocation that resulted in a patient experiencing continuous GHJ dislocations secondary to involuntary violent muscular spasms and emphasize the role of the physical therapist’s differential diagnosis and clinical decision-making process in a patient following direct access referral. *Case presentation:* A professional 23-year-old cyclist presented to a physical therapist with spontaneous multidirectional dislocations to the right shoulder after the recurrence of trauma occurred during a recent cycling race. The dislocations do not occur at night, but occur during the day, randomly, and mostly associated with changes in the patient’s psychological conditions. Directly from the clinical history, the physical therapist identified a neuro-physiological orange flag as well as an orthopedic red flag and, therefore, decided it was appropriate to refer the patient to a neurologist. It was determined by the physical therapist to be a priority to focus on the patient’s neurologic status and then to evaluate the orthopedic problem. The neurological examination revealed a condition of spontaneous multidirectional dislocation associated with recurrent antero-posterior pain spasms of the shoulder joint. The neurologist prescribed medication. Following the second cycle of medication assumption, the patient was able to continue physiotherapy treatment and was referred to the orthopedic specialist to proceed with shoulder stabilization surgery. *Discussion and conclusion:* Currently, the diagnosis of this unusual clinical condition is still unclear. It is a shared opinion of the authors that the trauma during the past bicycle race awakened an underlying psychological problem of the patient that resulted in a clinical condition of weakness of all the structures of the shoulder, such that these spasms could result in multiple multidirectional dislocations.

## 1. Introduction

Cycling is a popular source of recreation, transportation, and physical activity for children and adults [1]. There are more than 800 million bicycles in the world, and this is twice the number of motor vehicles [2]. Bicyclists have 2.3 times as many fatalities and 1.8 times as many nonfatal injuries as motor vehicle occupants per 100 million person-trips. For every two million trips, 600 injuries and one crash fatality are estimated to occur [2,3]. With regard to the total number of sports injuries per year, cycling has the highest absolute number of injuries per year in the United States (US) population, followed by basketball and football, as reported by Silberman et al. for US players. Cycling also has been found to be the most common sports-related activity associated with injuries among children, aged 5–14 years, more than basketball, football, and playground equipment [4]. Cycling injuries can be classified into three distinct areas: Bicycle contact, traumatic, and overuse injuries [4]. Traumatic injuries occurred in cycling 48.5% of the time, while overuse injuries occurred 51.5%, as reported in one study in top-level professionals [5]. More than two-thirds of traumatic injuries occur in the upper limb and cyclists seem to be exposed to high traumatic risk during multiple cycling activities such as racing in a peloton, at high speeds, on various road surfaces, and different weather conditions [6]. Regarding injuries occurring during group riding, most injuries occur when riding in a pace line or trying to avoid crashing into other crashed riders [4]. Many traumatic injuries do not require surgery, but may result in head injury, abrasion, contusion, single and multiple concussions, road rash, and fractures [7,8]. The most common anatomic area involved is the upper quadrant and generally the upper limb or shoulder, especially the clavicle, followed by the wrist, ribs, and elbow [4]. Off-road cycling in events appears to cause more fractures, dislocations, and concussions than road cycling in riding events [5]. In particular, the most common injuries to the shoulder are to the acromioclavicular joint (ACJ), sternoclavicular (SC) dislocation, and glenohumeral joint (GHJ) with anterior and/or posterior dislocation [9].

### 1.1. Anterior Shoulder Dislocation

Anterior traumatic shoulder dislocation is a common musculoskeletal condition that physical therapists (PT) and orthopedic surgeons see in practice [9]. This joint is the most common articulation involved in episodes of dislocation, with a rate of 11.2/100,000 per year and an estimated prevalence of 2% in the general population [10]. The main cause of primary shoulder dislocation is trauma, as almost 95% of the first episodes of dislocation are derived from a major collision, landing on an out stretched arm, or by a sudden and violent motion of the shoulder, with a peak of shoulder dislocations occurring in the second and sixth decade of life [11]. Shoulder dislocations in athletes occur more frequently than in the general population and result in substantial morbidity and time lost from sport. GHJ instability, which represents both shoulder dislocations and subluxations, most often occurs in an anterior direction, with nearly three-fourths of anterior dislocations occurring in males [12]. Injury patterns of the shoulder in young athletes are often specific to the involved sport. Similar to other acute traumatic injuries of the shoulder, such as acromioclavicular separation and clavicle fractures, traumatic glenohumeral dislocation is more common in contact sports, such as football, rugby, and lacrosse [13].

### 1.2. Comorbidities Associated with Shoulder Dislocation

A number of shoulder girdle injuries in sport are associated with acute anterior GHJ dislocations: Neurological diseases following reduction and either a rotator cuff tear or a greater tuberosity fracture. In terms of prevalence, traumatic anterior shoulder dislocation is common as well as brachial plexus injury [14] and axillary and musculocutaneous nerves injuries [15]. Rotator cuff tear, greater tuberosity fracture, or neurological deficit following primary anterior GHJ dislocation is greater than previously appreciated; these associated injuries may occur alone or in combined patterns [13].

### 1.3. Bicycle Injury Costs

Specific studies investigating the costs of managing an injury to competitive cyclists have not been conducted even though bicycling is an important mode of transportation in urban environments. 

For example, San Francisco has seen a 58% increase in the number of observed cyclists in 2009 alone [16]. Of all the bicycle-related injuries recorded at the San Francisco General Hospital, 41.5% were isolated cyclist impact injuries and 58.5% were from car impact injuries. The costs for assistance were significantly higher for car impact injuries with the total cost for isolated and impacted car impact injuries reached $12.6 and $17.8 million, respectively [16].

Differently, in Florida, an estimated 1.7 million youth have Medicaid insurance: The cost of sports injuries in youth was represented by $24 million for inpatient care and $87 million for emergency department care. Youth averaged $6039 for an inpatient visit and $439 for an emergency department visit in costs secondary to injuries from sports. Baseball, basketball, bike riding, American football, roller-skating/skateboarding, and soccer are sports with high costs for both emergency department patients and inpatients. Injuries from non-contact sport participants are few but can have high costs [17]. 

The aims of this case report are to report a rare clinical complication of GHJ anterior dislocation that resulted in the patient experiencing continuous GHJ dislocations secondary to involuntary violent muscular spasms and emphasize the role of the PT’s differential diagnosis and clinical decision-making process in a patient following direct access referral. To the authors’ knowledge, this is the first case report to describe this presentation.

## 2. Case Presentation

### 2.1. Patient History

The patient was a 23-year-old Italian male, professional cyclist, non-smoker, 190 cm tall and 82 kg, with a daily training load ranging from three to six hours, six days a week for an upcoming competition. The patient presented with no prior medical record but spontaneous and multidirectional multiple posterior dislocations of the right shoulder. In association with this complaint, the patient stated that he had widespread pain of the GHJ and paresthesias of the entire right hand, but predominantly on the palmar side of the fourth and fifth finger. Moreover, he complained about diffuse spasms (clonic manifestations) occurring around the shoulder, primarily of the pectoralis major and the biceps brachii muscles (Video S1). 

**Figure 1:**
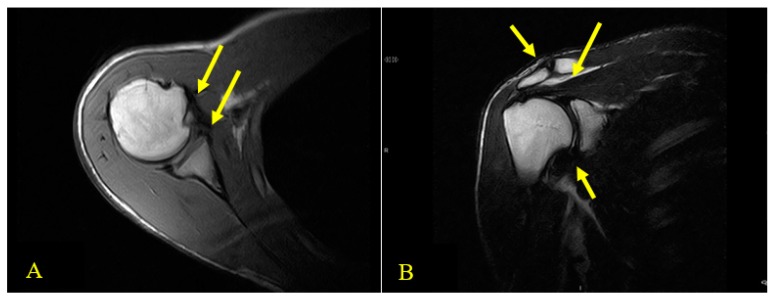
(**A**) and (**B**) Axial and coronal views of T1-weighted magnetic resonance imaging (MRI) scans. (**A**) Axial view of T1-weighted MRI. Two thick yellow arrows indicate inhomogeneity of the antero-inferior portion of the glenoid rim (bottom right), a McLaughlin lesion in the anterior aspect of the humeral head, and an extensive tear of the subscapularis tendon (top right). (**B**) Coronal view of T1-weighted MRI. Three thick yellow arrows indicate unevenness of the ACJ (top left), lesion of the pre-insertional bundles of the supraspinatus and fluid distension of the subacromial bursa (SAB) with diastasis of the glenoid bone (top right) and, slight thickening of the anterior capsule ligament (bottom right).

After a fall during a cycling race, the athlete went to a medical clinic in March 2017 and was diagnosed with subsequent dislocations of the right shoulder. The fall occurred following contact with a teammate during an international championship while riding on a paved road. The patient was not able to describe the exact traumatic mechanism but remembers his attempt to place his hands toward the ground to avoid the collision. The diagnostic investigation at the time, a magnetic resonance image (MRI), signaled the presence of “intra-articular effusion with non-homogeneity of the antero-inferior portion of the glenoid rim, bone marrow edema on the anterior surface of the humeral head, and an extensive lesion of the subscapularis tendon” (Figure 1). The anamnestic examination was highlighted by a numeric pain rating scale (NPRS) [18] of 6/10 (while the patient was at rest), widespread tenderness on the entire joint complex, and positive clinical tests (posterior drawer test, sulcus sign, load and shift, and apprehension test). The patient did not report further trauma to the joint until May 2018, when, during normal work activities (while lifting a box onto a shelf that was above shoulder height), the patient experienced another posterior dislocation of his right shoulder. After this dislocation, the number of episodes of dislocation during limb movements in normal daily activities of living increased in frequency and intensity. Subsequently, spasms of the joint complex followed these episodes and became more frequent with multiple spontaneous and multidirectional dislocations of the shoulder (Video S2). 

In June 2018, he returned to the clinic, complaining of a worsening of his clinical presentation and symptoms, characterized by an increase in spasmodic muscles surrounding his right shoulder, resulting in spontaneous multidirectional dislocations. The patient stated that these muscle spasms were associated with constant pain that worsened after the event and caused significant instability, weakness, and temporary tingling of the entire hand that was predominately located in the fourth and fifth finger. The spasms did not occur at night during sleep but appeared randomly and without warning throughout the day without apparent cause according to the patient. The patient also noted that the spasms were influenced by the patient’s mental state (with a reported increase of episodes and worsening following family disputes).

### 2.2. Physical Examination and the Physical Therapist’s Diagnosis

By analyzing the patient’s clinical history, family history, and the assessment scales, the PT identified various red and orange flags [19] during the medical history and through analysis of the patient’s disability of the arm, shoulder, and hand (DASH) rating scale (depression, anxiety, and somatoform disorders). At the initial evaluation, the PT observed that the right shoulder was in an anterior and internally rotated position. The patient’s right scapula was also abducted and internally rotated. Muscular trophism was reduced in front of the contralateral side: Deltoid, biceps, and pectoral were hypotrophic. No differences in temperature, skin color, or swelling were appreciable and there were no scars present.

The PT had no opportunity to perform any clinical tests as the patient’s passive and active movements immediately set off multidirectional joint dislocations and any type of stimulation increased the spontaneous muscle spasms. Three assessment scales were used by the PT to frame pain presentation (NPRS), disability/psychosocial factors (DASH) [20], and instability patterns (Western Ontario shoulder instability index (WOSI)) [21].

The patient’s NPRS was 8/10. The DASH score was used to evaluate the disability/psychosocial factors: This scale evaluates the patient’s ability to perform a range of daily, work-related, and sports/recreational activities. The DASH reported a 74.6% disability for daily life activities (ADL) and a 100% disability for work/recreational activities. 

The WOSI was used to evaluate the pathology-specific life quality for the patient with shoulder instability and the patient performed at 5.43%.

## 3. Intervention

### 3.1. Initial Physical Therapist’s Intervention

The PT referred the patient to the orthopedic specialist who recommended medical and diagnostic tests in addition to the X-rays (performed in the emergency room during one of the spasm episodes with dislocation), including another MRI and a computed tomography scan (CT scan). 

The MRI exam revealed several abnormal findings including an abnormal ACJ, lesion of the pre-insertional bands of the supraspinatus, fluid distension of the subacromial bursa (Sab) with glenoid diastasis, and a slight capsulo-ligamentous thickening on the anterior side. The CT scan and X-ray did not reveal any clinically relevant findings. The orthopedic examinations reported a diagnosis of multidirectional instability and the specialist recommended physical therapy focused on global muscle strengthening prior to surgery. The orthopedic surgeon did not report on the spasmodic related episodes. 

### 3.2. Neurology Referral

In consideration of the patient’s clinical presentation and his inability to tolerate any type of physiotherapy treatment, the PT considered it appropriate to further evaluate the associated “spasm” aspect. Therefore, the patient was referred to a neurologist in order to have a complete picture of the condition and to be able to follow the best management approach. The clinical consultation with the neurologist was performed in July 2018 and he noted a condition of multidirectional spontaneous dislocations associated with recurrent antero-posterior painful spasms of the shoulder joint. He advised reinforced physiotherapy in order to maximize the mechanisms of restraining the elusive humeral head as a function for future surgical procedure. The neurologist prescribed Gabapentin two times a day and Thiocolchicoside (TCC) and Alprazolam medications to eliminate episodes of spontaneous spasms. 

The effects of the medications started to act from the third day of recruitment, both in terms of a reduction of the number of attacks (one attack during the day) and in terms of intensity (less violent spasms and no back block) and duration (a few seconds). After one week of medications, the patient reported a cessation of spontaneous spasms.

### 3.3. Rehabilitation Phase

From that moment, the PT started a rehabilitation program addressing the overall joint reinforcement, progressing from isometric exercises (arm by side in internal/external rotation) to graded exposure to increase the intensity of the exercises (concentric isotonic exercises, recruitment of the scapula stabilizers, and motor control training with laser position sensor, Fluiballs, and closed kinetic chain quadruped exercises on the ball). The overall program was focused on pain-free exercises and avoidance of dislocations. On August 9th, the patient discontinued the medications and reported only one episode of spasm, which was associated with a family argument with his girlfriend. The spasm episode was described to be of short duration and low intensity. During the physiotherapy sessions, brief spasmodic episodes occurred when the patient was stimulated with ischemic compression techniques, both on the shoulder and at a distance (in sham modality), and also when the PT light touched with the fingertips in sham mode along various parts of the body (gluteus, gastrocnemius, left epicondyle).

### 3.4. Additional Presentation of Spasm

**Figure 2:**
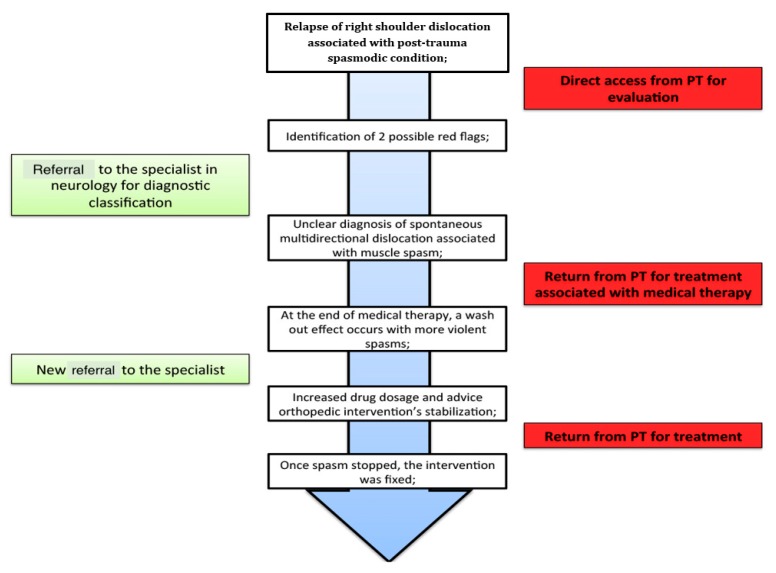
Timeline: The patient’s timeline for the multidisciplinary management.

On August 20th, 11 days after not taking medications, the spasms re-appeared without any apparent cause (no declared stressful events) with consequent multidirectional multiple painful dislocations with posterior block, that forced the patient to hospitalization. Upon reporting to the hospital, the physicians needed to administer Ketorolac and Apomorphine to reduce the posterior dislocation. Following this second event, the PT was contacted by the patient’s sister and informed that in the patient’s family medical history there was a condition of psychological disorders characterized by temper tantrums associated with temporary loss of consciousness and bipolar disorder. Therefore, the PT contacted the neurologist by telephone, informing him of this new health status information; moreover, the therapist was unable to continue rehabilitation secondary to the frequency, duration, intensity, and general worsening of the spasms. At this point, the physician prescribed 15 days of Gabapentin (twice a day) and then another 15 days once a day (in the evening) to reduce the wash-out effect. This time the pharmacological intake delayed its effects with respect to the first cycle, so that the PT resumed the rehabilitative work as soon as the spasms ceased, on August 29th. The PT noted that the spasms recurred occasionally during changes in mood and were linked to family arguments. On September 6th, the patient reduced the pharmacological treatment of Gabapentin to once a day; four days after the planned pharmacological decrease and, as noted by the patient, during a daily life problem, spasms of high intensity, frequency, and duration recurred and this time was associated with a temporary loss of consciousness. Secondary to these patient complaints, the PT consulted the neurologist, who advised the continuation of full-dose drug therapy (two times a day) and advised for a surgical stabilization procedure (Figure 2). On the fourth day of medical therapy, the patient’s spasms were reduced, and the patient resumed rehabilitation. 

### 3.5. Second Medical Intervention 

The neurologist identified the problem as recurrent antero-posterior spasms that resulted in associated spontaneous multidirectional dislocations and recommended the continuation of physiotherapy associated with drug therapy (Gabapentin 100 mg, twice a day dosage, morning and evening; TCC 8 mg) to break down the clonus. The neurologist did not provide a precise diagnosis: Using the term “spasm”, he only recognized an objective clinical sign observable during the evaluation. Unfortunately, after 18 days of medications and the subsequent wash-out effect (occurred 11 days later), the spasms recurred, increased in intensity and frequency, secondary to family arguments. The neurologist then prescribed another 15 days of medical therapy with Gabapentin 100 mg and TCC 8 mg twice a day followed by 15 more days of medications once a day to contain the wash-out effect; unluckily even during this second cycle of drug assumption the spasmodic phenomenon recurred at the time of the reduction of pharmacological therapy (always in association with mood changes). These last events led the neurologist to return to a twice a day intake and to plan joint stabilization surgery as soon as possible.

### 3.6. Surgery and Rehabilitation

**Figure 3:**
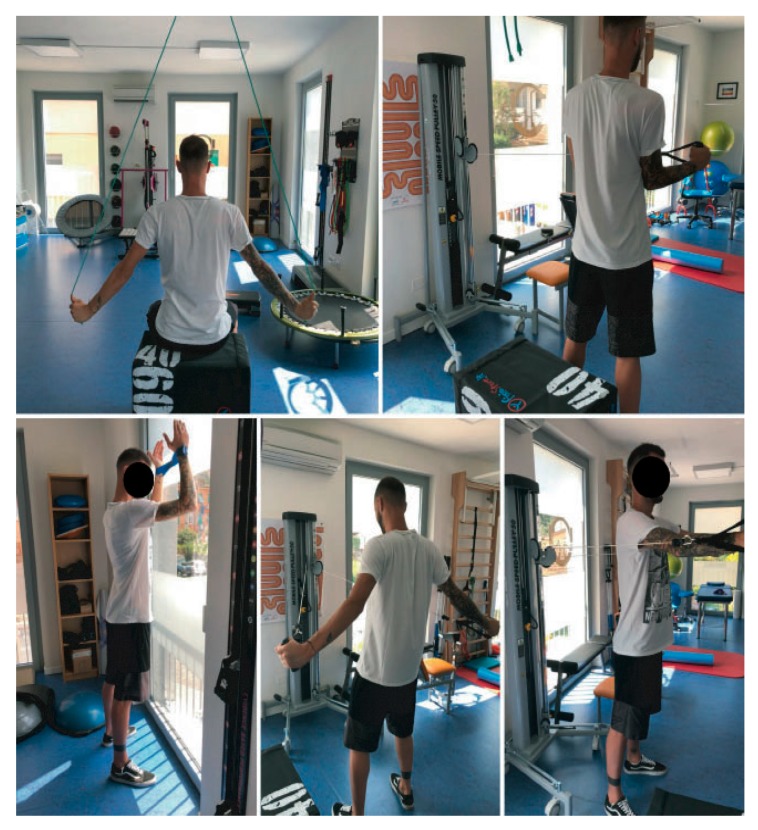
Images showing the patient performing a series of isometric, concentric, and eccentric isotonic exercises. Exercises were graded according to the patient’s ability to handle an increasing difficulty of task focused on the stability of the glenohumeral joint (GHJ) and the scapulothoracic joint (STJ).

The patient had surgery on 25 October 2018 with posterior arthroscopic capsuloplasty. On the advice of the orthopedic specialist, the patient was immobilized with a brace for 30 days and continued the pharmacological treatment with the same dosage, in order to monitor the spasms (they did not appear during the entire period of hospitalization and immobilization). Starting from the 30th day of bracing, the patient began the rehabilitation program, as suggested. However, starting from cautious techniques of passive mobilization in abduction, on the scapular plane, the spasms immediately reappeared, predominantly on the pectoralis major, deltoid, and biceps brachii muscles, characterized by low intensity and duration, and frequency linked to the movement. Therefore, the PT instantly called the referring orthopedist, who advised the PT to interrupt rehabilitation in order to avoid these excessive contractions or clonus—as these uncontrolled spasms could harm the results of the surgical intervention. It was also recommended to the patient to keep in touch with the neurologist to update him on the evolution of the clinical conditions. After a telephone interview with the neurologist, the patient was then asked to add Gabapentin 100 mg in the middle of the day, cyclobenzaprine 10 mg (one tablet, twice a day, for one week). The patient immediately started the new drug dosage; unfortunately, after a few days, side effects appeared (it is still uncertain and unclear whether these effects were determined by the increase of Gabapentin assumption or the addition of cyclobenzaprine), characterized by insomnia, anger attacks, loss of sexual libido, and hair loss. These unwanted consequences, added to his relative’s discouragement, pushed the patient to independently suspend drug therapy on 4 January 2019. Physiotherapy was not interrupted based on the patient’s decision, instead. Physiotherapy then continued essentially with proper attention to the healing processes and in order not to elicit and/or accentuate spasms. Techniques of mobility and muscular strength were associated with manual therapy techniques (tractions, glides, and soft-tissue mobilization of the scapular region); therapeutic exercise was introduced to allow the patient to carry out the main activities of daily life. The rehabilitation program continued for about one month and a half with a frequency of two sessions a week. Due to the persistent presence of uncontrolled spasms that showed themselves with free-movements and/or with pain, the PT could not increase the load and intensity of the exercise therapy necessary to obtain functional improvements (mostly from the point of view of range of motion (ROM) and strength) (Figure 3). At this point, the patient interrupted the physiotherapy program; he was able to perform 90° of active anterior flexion, 90° of active abduction, 60° of active external rotation, 40° of active internal rotation, all associated with an important scapular dyskinesis (characterized by the presence of an elevated, abducted, and upward-rotation-positioned scapula). At the end of the post-surgery physiotherapy cycle, the outcome measures proposed in the care of the patient (NPRS, DASH, and WOSI) were repeated and recorded the following scores: NPRS 1/10; DASH 15% disability with regard to ADL, 37.5% for work activities, and 100% for sports activities; and WOSI registered a rate of 1.25%. These granted movements allowed him to return to the performance of all the activities of daily life and return to work, but still no return to competitive sport was achievable, at least in the short-/medium-term prognosis.

## 4. Discussion

This case report was written following items of the CARE checklist [22]. The discussed clinical case aims to focus attention on the key role of the specialized PT to be able to recognize red flags and refer the patient to the specialist doctor, as well as to identify the appropriate specialist to reduce the time of clinical management. Moreover, the authors highlight the importance of multidisciplinary teamwork in the management of a patient with shoulder instability and high functional demand (professional cyclist). To the best of the authors’ knowledge, after shoulder dislocation, complications related to involuntary muscle spasms have not been reported in the literature. 

These spasmodic phenomena influenced the whole rehabilitation process, resulting in some difficulties in the therapeutic approach and during the rehabilitation period. Each therapeutic proposal, passive or active, has been adapted in order to avoid not only structural problems related to shoulder dislocation, but also the onset of phenomena of the central nervous system (spasms). The PT tried to obtain the entire recovery process in a way to avoid treatment complications and to obtain an optimal level of compliance from the patient; the patient’s perception of global management meant that a PT/patient relationship was established immediately. Secondly, each phase of the rehabilitation process was based on literature reviews and solid expert opinions, as written in the latest studies by Cools et al. [23] and Warby et al. [24].

Most recently, the evaluation of orange flags associated with psychiatric disorders (e.g., depression, anxiety, and somatoform disorders) and yellow flags related to maladaptive pain coping strategies (e.g., kinesiophobia, catastrophic thoughts, or fear avoidance behavior) have become fundamental in physical therapy practice. However, the prevalence of psychiatric disorders in patients with movement disorders has increased. In the profession of physical therapy, patients with psychiatric conditions, must be referred for medical interventions such as pharmacological and/or behavioral therapy to facilitate the patient’s rehabilitation process [9,10,11]. Recognition of red, orange, and yellow flags and referral to the appropriate health care provider are essentials to improve patient outcomes. Generally, the management of a patient with a traumatic shoulder instability is surgical. Conservative physical therapy should be based on the recovery of the patient’s motor control and the strength of the glenohumeral soft tissues together with the restoration of the best functional scapular position; the rehabilitative approach should be encouraged for at least 12 weeks [24]. This patient suffered an episode of recurrent posterior traumatic dislocation during a cycling race with the subsequent development of complications, probably related to psychological factors. In this patient’s case, knowing his past medical history and specifically his past psychological history may have influenced the efficiency of medical referral and to whom this patient was referred. The patient’s frequent violent muscle spasms caused a painful multidirectional dislocation with a non-reducible posterior dislocation. The PT’s responsibility is to structure an extensive and accurate medical history collection that includes aspects of recent and past medical history, family history as well as to collect/identify conditions with red–orange–yellow flags to support the referral to another health professional for aspects that lie outside the realm of the PT’s scope of practice. In this case report, the PT consulted an orthopedic specialist to evaluate the concerns of posterior dislocation and a neurologist to assess the clonic manifestations (spasms). The appropriate teamwork between the PT, the orthopedist, and the neurologist led to the resolution of the patient’s problem. The PT’s ability to quickly identify the primary and working problem in close relationship with the appropriate medical team (neurologist in the first place and secondarily orthopedic) reduced the patient’s recovery time and, consequently, the costs incurred by the patient.

The diagnosis of this unusual clinical condition is still unclear. It is a shared opinion of the authors that the trauma of the bicycle race triggered an occult psychological condition that influenced the patient presentation and led to multiple multidirectional shoulder dislocations.

The sports PT plays a fundamental role in both preventive and post-injury management for the elite athlete and works as an important part of a team of health care professionals to promote the well-being of the athlete to maximize sporting performance and prevent any type of injury. The PT may act as a manager to address ongoing situations as they arise, which reduces the timing (and therefore the relative costs both in economic terms and in terms of damage from the athlete’s absence in a competition) and the likelihood of a recurrence. In this case report, the authors highlight the PT’s role in a differential diagnosis. The patient in this case report should have been treated for free at the rehabilitation center of the sports club to which he belongs, but he decided to use personal insurance and pay the rehabilitation expenses for around EUR 3000 on his own.

Previous literature on a patient like the one in this case report does not exist. Carroll et al. report a patient case of dystonia of the shoulder after trauma in the US military [25]. This patient was not diagnosed by the neurologist with a clear diagnosis, but a clinical and objective symptom of spasm was associated with psychosomatic components. Moreover, in the comparison between the two cases, the authors note the differences in occupation, the first a US soldier, the second a professional cyclist.

## 5. Conclusions

This paper emphasizes the role of the PT in direct access to identify those patients that are appropriate for referral to another health care provider. 

Authors have also tried to highlight how a structural problem of the GHJ may underlie an intrinsic psychological framework as a triggering cause, hence the importance of the specialized PT to identify the problem at the base and thus able to carry out a correct referral; moreover, it is important to underline how the teamwork between several health figures (PT–neurologist–orthopedist) allowed the patient to get back to an excellent life condition.

## Supplementary Materials

The following are available online at https://www.mdpi.com/1010-660X/55/9/529/s1, Video S1: The patient is lying in a supine position during the initial physical therapist evaluation via direct access. The physical therapist’s hand monitors the clonic manifestation at the GHJ resulting in spontaneous multidirectional dislocations, Video S2: The patient is sitting during one of the clonic manifestations that provoked a multidirectional dislocation.

## Abbreviations

US	United States
ACJ	Acromioclavicular joint
SC	Sternoclavicular
GHJ	Glenohumeral joint
PT	Physical therapist
MRI	Magnetic resonance image
NPRS	Numeric pain rating scale
DASH	Disability of the arm, shoulder, and hand
WOSI	Western Ontario shoulder instability
ADL	Activities of daily living
CT scan	Computed tomography scan
SAB	Subacromial bursa
ROM	Range of motion
STJ	Scapulothoracic joint
TCC	Thiocolchicoside
